# The misuse of distributional assumptions in functional class scoring gene-set and pathway analysis

**DOI:** 10.1093/g3journal/jkab365

**Published:** 2021-10-25

**Authors:** Chi-Hsuan Ho, Yu-Jyun Huang, Ying-Ju Lai, Rajarshi Mukherjee, Chuhsing Kate Hsiao

**Affiliations:** 1 Division of Biostatistics and Data Science, Institute of Epidemiology and Preventive Medicine, National Taiwan University, Taipei 10055, Taiwan; 2 Department of Biostatistics, Harvard University, Boston, MA 02115, USA; 3 Bioinformatics and Biostatistics Core, Center of Genomic Medicine, National Taiwan University, Taipei 10055, Taiwan

**Keywords:** association study, gene expression, gene set analysis, multivariate normality test, pathway analysis

## Abstract

Gene-set analysis (GSA) is a standard procedure for exploring potential biological functions of a group of genes. The development of its methodology has been an active research topic in recent decades. Many GSA methods, when newly proposed, rely on simulation studies to evaluate their performance with an implicit assumption that the multivariate expression values are normally distributed. This assumption is commonly adopted in GSAs, particularly those in the group of functional class scoring (FCS) methods. The validity of the normality assumption, however, has been disputed in several studies, yet no systematic analysis has been carried out to assess the effect of this distributional assumption. Our goal in this study is not to propose a new GSA method but to first examine if the multi-dimensional gene expression data in gene sets follow a multivariate normal (MVN) distribution. Six statistical methods in three categories of MVN tests were considered and applied to a total of 24 RNA data sets. These RNA values were collected from cancer patients as well as normal subjects, and the values were derived from microarray experiments, RNA sequencing, and single-cell RNA sequencing. Our first finding suggests that the MVN assumption is not always satisfied. This assumption does not hold true in many applications tested here. In the second part of this research, we evaluated the influence of non-normality on the statistical power of current FCS methods, both parametric and nonparametric ones. Specifically, the scenario of mixture distributions representing more than one population for the RNA values was considered. This second investigation demonstrates that the non-normality distribution of the RNA values causes a loss in the statistical power of these GSA tests, especially when subtypes exist. Among the FCS GSA tools examined here and among the scenarios studied in this research, the N-statistics outperform the others. Based on the results from these two investigations, we conclude that the assumption of MVN should be used with caution when evaluating new GSA tools, since this assumption cannot be guaranteed and violation may lead to spurious results, loss of power, and incorrect comparison between methods. If a newly proposed GSA tool is to be evaluated, we recommend the incorporation of a wide range of multivariate non-normal distributions or sampling from large databases if available.

## Introduction 

Gene-set analysis (GSA) is one of the standard procedures used in biomedical research when interest lies in the evaluation of certain biological functions, such as pathways, or of the collective effect of a set of genes on disease status, phenotype, or other response variables (Hirschhorn 2009; Mooney *et al.* 2014). The development of the methodology for performing GSA has attracted much attention in the biostatistics and bioinformatics communities in recent decades. Most GSA methods are applied to pathways as a way of evaluating and testing pathway association. Findings in GSAs can provide a better understanding of disease mechanisms and identify potential treatment targets, particularly for complex diseases (Subramanian *et al.* 2005; Hirschhorn 2009; Mooney *et al.* 2014). Results from the procedures can save time, cost, and effort in translational studies.

When used for pathway analysis, it is common to categorize GSA methods into three classes: Over representation analysis, functional class scoring (FCS), and pathway topology methods. Several studies have reviewed and compared these different GSA categories (Goeman and Buhlmann 2007; Ackermann and Strimmer 2009; Gatti *et al.* 2010; Khatri *et al.* 2012; Maciejewski 2014; de Leeuw *et al.* 2016). The over representation analysis approach is concerned with determining if the differentially expressed (DE) genes are *overrepresented* (or *enriched*) in a candidate gene-set/pathway; that is, if the number of DE genes in the set is beyond chance alone. When this is the goal, an over representation analysis test is carried out based on the hypergeometric distribution, chi-square test, or Fisher’s exact test (Boyle *et al.* 2004). Whether the RNA values follow a normal distribution does not affect the over representation analysis test in the step of enrichment evaluation. The over representation analysis approach is intuitive and is still a widely used procedure to screen for potential targeted pathways from a list of known ones, thus it is often called a knowledge-based procedure. A major limitation of over representation analysis, however, is that it ignores the relationship among genes in the set of interest and considers them equally important.

The FCS approach aims to examine the collective and coordinated effect of the gene-set/pathway. It calculates a score at the pathway level based on a statistic evaluated at each individual component gene, whether it is DE or not. Most FCSs consider the score a weighted sum of the gene-level statistics, where the weight usually represents the interrelationship among the genes. The advantages of FCSs are the inclusion of genes that may not be significant at the single gene level, the incorporation of the dependence among genes, and the potential to compare contributions of different pathways. More discussion of various FCS methods is included in Section 3. The third category, the pathway topology methods, such as SPIA (Draghici *et al.* 2007), NetGSA (Shojaie and Michailidis 2009), and NetworkHub (Chang *et al.* 2020), all involve a known topological structure of the genes, and therefore can be applied only if the structure of the set of genes is already determined. This limits the application of pathway topology methods to established biological pathways. Consequently, it becomes difficult to evaluate their performance with simulation studies since the biological structure is required as input in pathway topology methods.

Several review articles have documented the evolution of GSA tools (Ackermann and Strimmer 2009; Maciejewski 2014; de Leeuw *et al.* 2016), with emphasis on their differences, assumptions, and statistical properties; some have also addressed issues that arise in GSA, including the lack of consideration of linkage disequilibrium (or correlation) between genes (Goeman and Buhlmann 2007; Gatti *et al.* 2010; Lin *et al.* 2018), the choice of null hypothesis (competitive or self-contained tests) (Goeman and Buhlmann 2007; Maciejewski 2014; de Leeuw *et al.* 2016), and the influence of the proportion of associated genes in the set (Maciejewski 2014; de Leeuw *et al.* 2016). A few studies have expressed doubts about the distributional assumptions made about the data (Kerr *et al.* 2000; Konishi 2004; Zyla *et al.* 2017). Whether or not the transcriptomic data are normally distributed has not been resolved, even though it has been noted that heterogeneity in the profiling values may exist due to disease subtypes (Konishi 2004; Zyla *et al.* 2017; Ho 2018; de Torrenté *et al.*, 2019; Liu *et al.* 2019; Kerr *et al.* 2000). Nevertheless, no study that we are aware of has investigated the impact of distributional assumptions on the conclusions of GSA when the gene-set exerts a certain molecular function or activity.

The commonly adopted distributional assumptions for gene expression data are the multivariate normal (MVN) or log-normal distribution. Despite doubts about this assumption, these distributions are frequently used to generate expression values in simulation studies when evaluating the performance of a newly proposed GSA tool, especially those GSAs categorized as FCS methods. In other words, many GSA tools inherently assume that the gene expression data are normally or log-normally distributed, or that the GSA tests are robust to the distributional assumptions. Since the normality assumption may not be true or guaranteed (Kerr *et al.* 2000; Konishi 2004; Zyla *et al.* 2017), such simulation-based assessment of GSA tools may be incomprehensive, leading to possibly spurious findings.

Whether or not the assumed distribution is appropriate may affect the applicability of GSA methods. The performance in terms of statistical power, for instance, may be affected when the data deviate from normality. If the power of the GSA tests is reduced when the RNA values follow a non-normal distribution, then it is possible that the gene-set/pathway associated with the response variable cannot be identified. To what extent the change in performance corresponds to the deviation requires further examination. The GSA methods that are mean-based (de Leeuw *et al.* 2016) may be able to guard against this issue if the size of the gene-set, the number of genes, is small enough relative to the number of samples for asymptotic normality to apply. For nonparametric GSA tests, it is not yet known how robust they are with respect to incorrect distributional assumptions.

Therefore, the two goals of this study are, first, to investigate the goodness-of-fit of the MVN distribution for the gene expression data and, second, to evaluate the performance of common FCS GSA methods in response to the deviation of the normality assumption. In the first part of this study, we will examine whether the parametric MVN distribution is a suitable assumption for gene expression values in the real world. To assess if the multi-dimensional data set at hand fits an MVN distribution, many tests have been proposed in the literature and several articles have presented comparisons and reviews of them (Thode 2002; Mecklin and Mundfrom 2005, 2004; Korkmaz *et al.* 2014; Chen and Xia 2019). Based on their recommendations, six tests are considered: the Mardia test (Mardia 1970), the Henze-Zirkler (HZ) test (Henze and Zirkler 1990), the Royston test (Royston 1992), the Fattorini (FA) test (Fattorini 1986; Lee *et al.* 2014), the TN test (Zhou and Shao 2014), and the Energy test (Székely and Rizzo 2005). These MVN tests will be applied to three types of RNA values, including microarray, RNA-sequencing (RNA-seq), and single-cell RNA-sequencing (scRNA-seq) data.

The second goal of this study is to compare the performance of several FCS GSA tools, with emphasis on the impact of the distributional assumption, based on data generated from MVN and non-normal distributions. The objective of these GSAs is to examine if a gene-set associates with a response variable, that is, if the gene-set shows a collective effect on the outcome variable, such as disease status, disease subtype or some continuous phenotypic value. Here, we consider specifically the GSA tools in the FCS group. In addition, we categorize the tools in this group into two different subgroups, parametric and nonparametric GSAs. The parametric GSA tools were developed mostly based on known statistical distributions and models, including the random-effects global test (Goeman *et al.* 2004), Hotelling’s statistic (Lu *et al.* 2005; Schafer and Strimmer 2005), and ROAST (Wu *et al.* 2010) in an MVN model, and the pathway activity score (*P*-score) in a logistic regression model (Lin *et al.* 2018). The nonparametric GSA methods mostly use the ranks of gene expression levels to reduce the heterogeneity in the absolute values across different genes. Several popular nonparametric methods are gene set enrichment analysis (GSEA) (Subramanian *et al.* 2005), the N-statistic (Baringhaus and Franz 2004; Klebanov *et al.* 2007; Glazko and Emmert-Streib 2009), and the Kolmogrov–Smirnov test for the mean vector and the Kolmogrov–Smirnov test for the covariance matrix (Rahmatallah *et al.* 2017). These GSA tools for association studies will be investigated to evaluate their performance under different distributional assumptions that deviate from MVN.

## Materials and methods

### Multivariate normality tests of gene expression values

#### Data source and data management

To investigate if MVN fits gene expression data, three types of RNA values, including microarray, RNA-seq, and scRNA-seq data, are considered for examination. Twenty-two microarray data sets and the RNA-seq data were downloaded from the National Center for Biotechnology Information (NCBI) and The Cancer Genome Atlas (TCGA) websites, and the scRNA-seq data (Tucker *et al*. 2020) were downloaded from the Broad Institute Single Cell Portal. Many of the microarray gene expression data sets were from cancer [breast, colorectal, lung, ovarian, and glioblastoma (GBM)] patients; four sets were from cancer-free patients with chronic obstructive pulmonary disease (COPD), two of smokers and two of nonsmokers. The RNA sequencing data from the NCBI were collected from breast cancer patients and normal controls; and the single-cell RNA sequencing data were from human donors without any clinical evidence of cardiac dysfunction.

Each of the microarray data sets was processed with the Robust Multi-array method (R function *rma* in R package *affy*) and quantile normalization (R function *normalize.quantiles* in R package *preprocessCore*) for data processing. Next, considering that expression levels are affected by age, gender, ethnicity, cancer grade and stage, these downloaded data were stratified according to these factors, if available, and then the groups containing a larger number of samples were selected for the following analyses. For instance, in the first data set (Ni *et al.* 2010), only breast cancer patients who were in grades II and III and were also Malaysian Malays were selected. In the second data set (Sabates-Bellver *et al.* 2007), the 23 colorectal cancer patients with tumors found in the rectum or sigmoid colon were selected from a study originally containing 32 patients. In the GBM study in the Cancer Genome Atlas Research Network (2008), only male patients aged beyond 50 and diagnosed with one of the four subtypes of GBM were selected. Table  1 lists for the RNA array data the type of cancer or disease, the accession number in the NCBI data portal or TCGA identification, the source of the sample (tissue, cell, or peripheral blood), the platform (Affymetrix GPL96/HG-U133A, Affymetrix GPL570/HG-U133plus2, or multiple platforms from Affymetrix and Agilent) used for obtaining the expression levels, the sample type (paired or independent), the number of samples in each group, and the selection criteria used to select samples.

**Table 1:** Description of the 22 microarray RNA data sets considered for the MVN tests

	ID^*a*^	Source^*b*^	Platform	Paired^*e*^	No.	Selection criterion
Case						
Breast	GSE15852	T	GPL96	Y	24	Grades 2 and 3; Malay
Colorectal	GSE8671	T	GPL570	Y	23	Rectum and sigmoid colon
Lung_1^f^	GSE7670	T	GPL96	Y	21	Adenocarcinoma; female
Lung_2^f^	GSE19804	T	GPL570	Y	47	Adenocarcinoma; early stage; female
Lung_3^f^	GSE19188	T	GPL570	N	25	Adenocarcinoma; male
Lung_4^f^	TCGA-lusc	T	GPL96	N	49	Squamous cell; stage I; male; age > 50
Lung_5^f^	TCGA-lusc	T	GPL96	N	44	Squamous cell; stages II and III; male; age > 50
Ovarian_1^c^	TCGA-ov	M	GPL96	N	47	Grade 3; bilateral; stages III and IV; white
Ovarian_2^c^	TCGA-ov	M	GPL96	N	116	Grade 3; bilateral; stages III and IV; white
GBM_c^g^	TCGA-gbm	T	Multiple	N	66	Classical subtype; male; age > 50
GBM_m^g^	TCGA-gbm	T	Multiple	N	78	Mesenchymal subtype; male; age > 50
GBM_n^g^	TCGA-gbm	T	Multiple	N	47	Neural subtype; male; age > 50
GBM_p^g^	TCGA-gbm	T	Multiple	N	58	Proneural subtype; male; age > 50
Control						
Breast	GSE15852	T	GPL96	Y	24	Grades 2 and 3; Malay
Colorectal	GSE8671	T	GPL570	Y	23	Rectum and sigmoid colon
Lung_1^f^	GSE7670	T	GPL96	Y	21	Adenocarcinoma; female
Lung_2^f^	GSE19804	T	GPL570	Y	47	Adenocarcinoma; early stage; female
Lung_3^f^	GSE19188	T	GPL570	N	41	Adenocarcinoma; male
COPD_1^d^	GSE11906	T	GPL570	N	31	Healthy smoker; small airway; male
COPD_2^d^	GSE42057	B	GPL570	N	27	Gold stages 3 and 4; male
COPD_3^d^	GSE11906	T	GPL570	N	26	Healthy nonsmoker; small airway; male
COPD_4^d^	GSE42057	B	GPL570	N	22	Gold stage 0; male

The gene expression data are either from cancer patients (case), from subjects without cancer or from adjacent normal tissues (control). The selection criterion is the conditions that were used to select samples from the original public data.

a GEO accession number or TCGA identification.

b T for tissue or cell; B for peripheral blood; M for tissue or blood.

c The two groups are poor (Ovarian_1) *vs* good (Ovarian_2) prognosis group.

d The two groups (COPD_1 and COPD_2) are smokers and the two groups (COPD_3 and COPD_4) are nonsmokers from the COPD study.

e If the data are paired samples, then the case group contains cancer tissue and control contains the adjacent normal tissue.

f Lung_1 is nonsmall cell lung carcinoma (NSCLC) patients with adenocarcinoma, Lung_2 and Lung_3 only specify NSCLC, Lung_4 and Lung_5 are NSCLC patients with squamous cell carcinoma.

g The four groups are the different subtypes, GBM_c for classical, GBM_m for mesenchymal, GBM_n for neural, and GBM_p for proneural, respectively.

The RNA-seq values were obtained from a study of pure high-grade ductal carcinoma in situ containing 25 patients and 10 normal controls (Abba *et al.* 2015). The normalized read counts of the expression values from 16,532 genes can be downloaded from the NCBI (accession number GSE69240) website. The expression values for the following analysis are on a logarithmic scale with base 2.

The scRNA-seq data were collected from seven donors who did not have overt cardiac disease (Tucker *et al.* 2020). This study sequenced 287,269 nuclei from normal heart tissue of the four chambers (left atrium, left ventricle, right atrium, and right ventricle) and identified 9 major cell types and more than 20 subclusters of cell types within the human heart. The grouping of these subclusters will be retained in the following analysis to reduce heterogeneity across clusters.

#### Selected statistical methods for MVN tests

Six methods in three categories are considered for the MVN test. The first category includes two tests based on the distance measure between the *p*-dimensional data and the *p*-variate normal distribution, the Energy test and the HZ test. The distance measure used in Energy is the Euclidean distance and in HZ it is the expected distance between two characteristic functions. The second category contains three tests which are all multivariate extensions of the univariate SW test: the Royston test which combines the SW statistic from each of the *p* coordinates, and the FA and TN tests which are based on projection of the multivariate data onto a one-dimensional point, where FA and TN utilize different statistics of the projected values. The test in the third group is the traditional Mardia test based on multivariate skewness or kurtosis. We consider any violation in either one as a deviation from MVN and therefore the Mardia test was applied here to test if either the skewness or kurtosis test reached significance at the 5% nominal level. Some background information about the six MVN tests is provided in Supplementary File S1.

All the tests were carried out in R, with the function *mvnorm.etest* in the R package *energy* for the one (multivariate) sample Energy test (Székely and Rizzo 2017), with the function *mvn* in the R package *MVN* for the HZ, Royston, and Mardia tests (Korkmaz *et al.* 2014) and with the functions *faTest* and *mvnTest* in the R package *mvnormalTest* for the Fattorini FA and TN tests (Lee *et al.* 2014), respectively.

#### Selected pathways and gene-sets for MVN tests

To test the gene sets for MVN, 10 signaling pathways from the Kyoto Encyclopedia of Genes and Genomes (KEGG) were selected deliberately. These signaling pathways were constructed based on interacting molecules involving specific biological functions, where the gene nodes in the pathway network (gene-set) would be expected to exert some correlation. Such pathways targeted by GSA therefore are the focus of our examination of the normality test. The 10 signaling pathways defined in KEGG are the p53, mTOR, Jak-STAT, PI3k-Akt, Wnt, ErbB, MAPK, RAS, TGF-β, and TNF pathways, which contain 60, 69, 32, 85, 76, 47, 115, 69, 58, and 82 gene nodes, respectively. These have been reported to associate with one or more of the cancers considered here. For example, the p53 pathway was reported to show association with breast (Gasco *et al.* 2002; Bertheau *et al.* 2008; Walerych *et al.* 2012), colorectal (Li *et al.* 2015), lung (Mitsudomi *et al.* 2000; Shtivelman *et al.* 2014), ovarian (Bernardini *et al.* 2010; Hayano *et al.* 2014), and GBM (Cancer Genome Atlas Research Network 2008; Jung *et al.* 2019) cancers. Association of the other pathways such as mTOR, Jak-STAT, PI3k-Akt, Wnt, RAS, and TGF-β have been reported as well (Park and Kim 2007; Cancer Genome Atlas Research Network 2008; Eroles *et al.* 2012; Shtivelman *et al.* 2014; Slattery *et al.* 2014; Lin *et al.* 2018; Jung *et al.* 2019). For the COPD studies, the pathways reported to associate with the disease or smoking status include Jak-STAT (Bahr *et al.* 2013; Yew-Booth *et al.* 2015; Nicholson *et al.* 2016), PI3k-Akt (Marwick *et al.* 2010; Kim *et al.* 2011; Bahr *et al.* 2013; Mercado *et al.* 2015), and mTor (Bahr *et al.* 2013; Mercado *et al.* 2015).

The expression values of genes in the above pathways were extracted from microarray RNA and RNA-seq for the MVN tests. For the scRNA-seq data, since most scRNA-seq data are sparse, we selected the top 100 most variable genes for the MVN tests so that the inversion of the covariance matrix, calculated in some of the tests, would not be singular. The MVN tests were carried out for each of the four chambers and each of the subclusters, since the single-cells identified in the same chamber or subcluster were considered function similarly and thus contain less variability (Tucker *et al.* 2020).

### Evaluating the impact of the distributional assumption

#### Parametric and nonparametric GSA tools

Among the FSC GSA tools we considered here, the first group of them contains four parametric tests: the global test (Goeman *et al.* 2004), Hotelling’s statistic (Schafer and Strimmer 2005; Lu *et al.* 2005), ROAST (Wu *et al.* 2010), and the pathway activity score (*P*-score) in the logistic regression model (Lin *et al.* 2018). The global test examines the existence of heterogeneity among the random gene effects under a parametric generalized linear model. The function *gt* in the R package *Globaltest* performs this test. The Hotelling’s *T*^2^ statistic compares the mean vectors of the expression profiles from subjects of different disease statuses with a modified estimate of the covariance matrix to incorporate possible correlation among genes. This test can be performed in the R package *Hotelling* with the function *hotelling.test*. The ROAST method is also constructed under the linear model. It combines the gene-level modified *t*-statistic to formulate a statistic for the gene-set, and determines the *P*-value, not via permutation of genes or samples, but by the rotation of the independent residual space to incorporate the intergenic correlations (Langsrud 2005). The function *roast* in the R package *limma* performs this test. The pathway activity score, *P*-score, is a statistic based on ranks of gene expression values and magnitudes of correlations (Lin *et al.* 2018). The score is computed for each sample and the enrichment analysis for association with the response variable is then tested in a logistic regression model under the case-control study design.

The second group contains nonparametric FCS GSA tests which utilize mostly the ranks of the expression values or some metric of the distance between genes. The first one we considered is the popular GSEA modified for the self-contained null hypothesis for a predetermined set of genes (Gentleman *et al.* 2008). It is nonparametric in the sense that no distributional assumption is made for the expression value and the test statistic is based on the ranks of the degree of association between the response and the individual gene. It assumes independence among gene expression profiles and utilizes rankings and nonparametric distance metrics to derive *P*-values. This test can be carried out with the function *gseattperm* in the R package *Category*. The second test, usually called the N-statistic, compares the Euclidean distance between multivariate observations from two response groups (Baringhaus and Franz 2004; Klebanov *et al.* 2007; Glazko and Emmert-Streib 2009). It follows the same rationale as in the energy test in previous sections for MVN tests, where a group of observations is compared against observations generated from a MVN distribution. Here for GSA, the focus is on the comparison between two groups of multivariate observations without distributional assumptions for either group. This test can be conducted with the function *eqdist.etest* in the R package *energy* (Székely and Rizzo 2017). The other two GSA tools considered here first rank the multivariate samples based on the minimum spanning tree and then use the multivariate Kolmogorov–Smirnov test under the self-contained null hypothesis to test for the difference in mean, or in variance (Rahmatallah *et al.* 2017). The R package adopted here is *GSAR* and the functions are *KStest* and *RKStest*, respectively. All the software and functions used in this study are listed in Table  2.

**Table 2:** Software (R package; function) used for assessing the performance of the FCS GSA tools

	R package	Function
Parametric GSAs		
Global test	*Globaltest*	*gt*
Hotelling’s *T*^2^ test	*Hotelling*	*hotelling.test*
ROAST	*limma*	*roast*
*P*-score	*base*	*glm*
Nonparametric GSAs		
GSEA	*Category*	*gseattperm*
N-statistic	*energy*	*eqdist.etest*
Kolmofrov–Smirnov test of mean	*GSAR*	*KStest*
Kolmogrov–Smirnov test of variance	*GSAR*	*RKStest*

#### Simulation setting I with a 1-component distribution per group (settings A and B)

To evaluate the performance of these GSA tools with respect to different distributions of the gene expression values, the following scenarios were considered. The first two are single-component models. Simulation setting A was designed under the *p*-dimensional MVN assumption (*P *=* *30), where both response groups, termed as *case* and *control* with 50 subjects in each group, were generated from MVN distributions with different mean vectors (the difference is denoted as $\underset{˜}{\Delta }$) and the same compound symmetry covariance matrix (with ρ* *=* *0.1).

Response group 1 (*control*) and 2 (*case*) in setting A:

$${\underset{˜}{y}}_{i}∼MVN(\underset{˜}{0},\Sigma =\left(\begin{array}{ccc} 1 & \cdots & 0.1\\ & \ddots & 0.1\\ & & 1 \end{array}\right)))vs.{\underset{˜}{y}}_{i}∼MVN(\underset{˜}{\Delta },\Sigma =\left(\begin{array}{ccc} 1 & \cdots & 0.1\\ & \ddots & 0.1\\ & & 1 \end{array}\right))$$

Setting B aims for a larger heterogeneity than A and the data were instead generated from multivariate t distributions (MVT) with 3 degrees of freedom, the same mean vectors as in A and a compound symmetry covariance matrix with ρ* *=* *0.5.

#### Simulation setting II with a 2-component mixture distribution per group (settings C and D)

In this setting, we consider distributions containing more variation than the above scenario by assuming a mixture of two distributions. In setting C, the expression values were randomly generated from a mixture of two MVN distributions, each with 50% weight. Specifically, in the *control* group, the mean vectors of the two component MVNs are the zero mean vector $\underset{˜}{0}$ and the vector of all-ones $\underset{˜}{1}$, respectively; while in the *case* group, the two mean vectors are $\underset{˜}{0}+\underset{˜}{\Delta }$ and $\underset{˜}{1}+\underset{˜}{\Delta }$, respectively.

Response group 1 (*control*) in setting C:

$${\underset{˜}{y}}_{i}∼0.5MVN({\underset{˜}{0}}_{30\times 1},\Sigma ={\left(\begin{array}{ccc} 1 & \cdots & 0.1\\ & \ddots & 0.1\\ & & 1 \end{array}\right)}_{30\times 30})+0.5MVN({\underset{˜}{1}}_{30\times 1},\Sigma ={\left(\begin{array}{ccc} 1 & \cdots & 0.5\\ & \ddots & 0.5\\ & & 1 \end{array}\right)}_{30\times 30}).$$

In setting D, the variability in each component is made even larger by assuming a MVT distribution with 3 degrees of freedom.

#### Simulation setting III in which each case group contains subtypes (settings E, F, G, and H)

In contrast to the previous scenarios where both *case* and *control* groups are from the same distribution but with different means, here we consider the scenario where the disease (case) group contains more than one component distribution. These situations can arise due to disease subtypes such as cancer grade, staging, and tumor tissue type (Kim *et al.* 2010). Examples include lung cancer with two major subtypes, small cell lung cancer (SCLC) and nonsmall cell lung cancer (NSCLC), and leukemia with four major subtypes, acute lymphoblastic leukemia, acute myeloid leukemia, chronic myeloid leukemia, and chronic lymphocytic leukemia. The therapeutic choice may differ according to different subtypes where such classification is usually performed clinically with immunohistochemistry (IHC). Therefore, in settings E and F, the distribution for the *case* group is a 2-component mixture of normal distributions (setting E) and a 2-component mixture of t distributions (setting F), respectively; whereas in settings G and H, it is a 3-component mixture distribution. Table  3 lists all the distributions considered in these simulation studies.

**Table 3:** Distributions used to generate expression data in the GSA analysis

	Distribution for the control group	Distribution for the case group	KL divergence^*a*^
Settings I: 1-component *vs* 1-component			
A	MVN($\underset{˜}{0},\Sigma_{1}$)	MVN($\underset{˜}{\Delta },\Sigma_{1}$)	3.38
B	MVT($\underset{˜}{0},\Sigma_{2}$)	MVT($\underset{˜}{\Delta },\Sigma_{2}$)	18.19
Settings II: 2-component *vs* 2-component			
C	0.5MVN($\underset{˜}{0},\Sigma_{1}$) + 0.5MVN($\underset{˜}{1},\Sigma_{2}$)	0.5MVN($\underset{˜}{0}+\underset{˜}{\Delta },\Sigma_{1}$) + 0.5MVN($\underset{˜}{1}+\underset{˜}{\Delta },\Sigma_{2}$)	14.30
D	0.5MVT($\underset{˜}{0},\Sigma_{1}$) + 0.5MVT($\underset{˜}{1},\Sigma_{2}$)	0.5MVT($\underset{˜}{0}+\underset{˜}{\Delta },\Sigma_{1}$) + 0.5MVT($\underset{˜}{1}+\underset{˜}{\Delta },\Sigma_{2}$)	23.13
Setting III: 1-component *vs* 2-component/3-component			
E	MVN($\underset{˜}{0},\Sigma_{1}$)	0.5MVN($\underset{˜}{0},\Sigma_{1}$) + 0.5MVN($\underset{˜}{\Delta },\Sigma_{1}$)	1.22
F	MVT($\underset{˜}{0},\Sigma_{2}$)	0.5MVT($\underset{˜}{0},\Sigma_{2}$) + 0.5MVT($\underset{˜}{\Delta },\Sigma_{2}$)	15.24
G	MVN($\underset{˜}{0},\Sigma_{1}$)	0.4MVN($\underset{˜}{0},\Sigma_{1}$) + 0.3MVN($0.5\times \underset{˜}{\Delta },\Sigma_{1}$) + 0.3MVN($\underset{˜}{\Delta },\Sigma_{1}$)	0.97
H	MVT($\underset{˜}{0},\Sigma_{2}$)	0.4MVT($\underset{˜}{0},\Sigma_{2}$) + 0.3MVT($0.5\times \underset{˜}{\Delta },\Sigma_{2}$) + 0.3MVT($\underset{˜}{\Delta },\Sigma_{2}$)	15.17

MVN stands for the MVN distribution and MVT stands for multivariate t distribution. The two covariance matrices adopted both have compound symmetry correlation structure where the correlation is 0.1 in $\Sigma_{1}$ and 0.5 in $\Sigma_{2}$, respectively. The KL divergence measures the distance between the distribution based on simulated data and the MVN $N_{p}$with zero mean vector and an identity covariance matrix. All vectors ($\underset{˜}{0}$, $\underset{˜}{\Delta }$, and $\underset{˜}{1}$) are 30-dimensional and matrices ($\Sigma_{1}$ and $\Sigma_{2}$) are 30 by 30.

a The sum of the KL divergence between the case and $N_{p}$, and the KL divergence between the control and $N_{p}$. KL divergence was computed with the function *KL.dist* in the R package *FNN*.

The use of mixture distributions for tumor subtypes incorporates the scenario of skewed distributions which are apparently non-normally distributed (Kim *et al.* 2010). To demonstrate the degree of deviation from normality, the Kullback-Leibler (KL) divergence is adopted to measure the *distance* between the distribution of the simulated data and $N_{p}$, a MVN distribution with a zero mean vector and an identity covariance matrix, in each setting. The values are listed in the right-most column in Table  3. Larger values of KL divergence imply a greater difference between the group with the simulated distribution and the group with a MVN distribution.

## Results

### Normality tests of gene expression values

Since MVN will not hold true for the distribution of any pathway if any subset of the pathway deviates from the normality assumption, we selected a subset of either 10 (if the number of samples exceeds 30) or 5 (if the number is below 30) genes from each pathway (for the array RNA and RNA-seq data) or from each chamber or subcluster (for the scRNA-seq data) and carried out the above six MVN tests. This procedure was repeated 1000 times and the rejection rate, the proportion $Q=\#\{p_{i}\leq 0.05\}/1000$ of replications in which the *P*-value $p_{i}\;(i=1,\ldots ,1000)$ was less than the nominal 0.05, was recorded. If the RNA values are in fact normally distributed, then the rejection rates Q’s would be around 0.05, since the *P*-values under the null hypothesis follow a uniform distribution.

**Figure 1:**
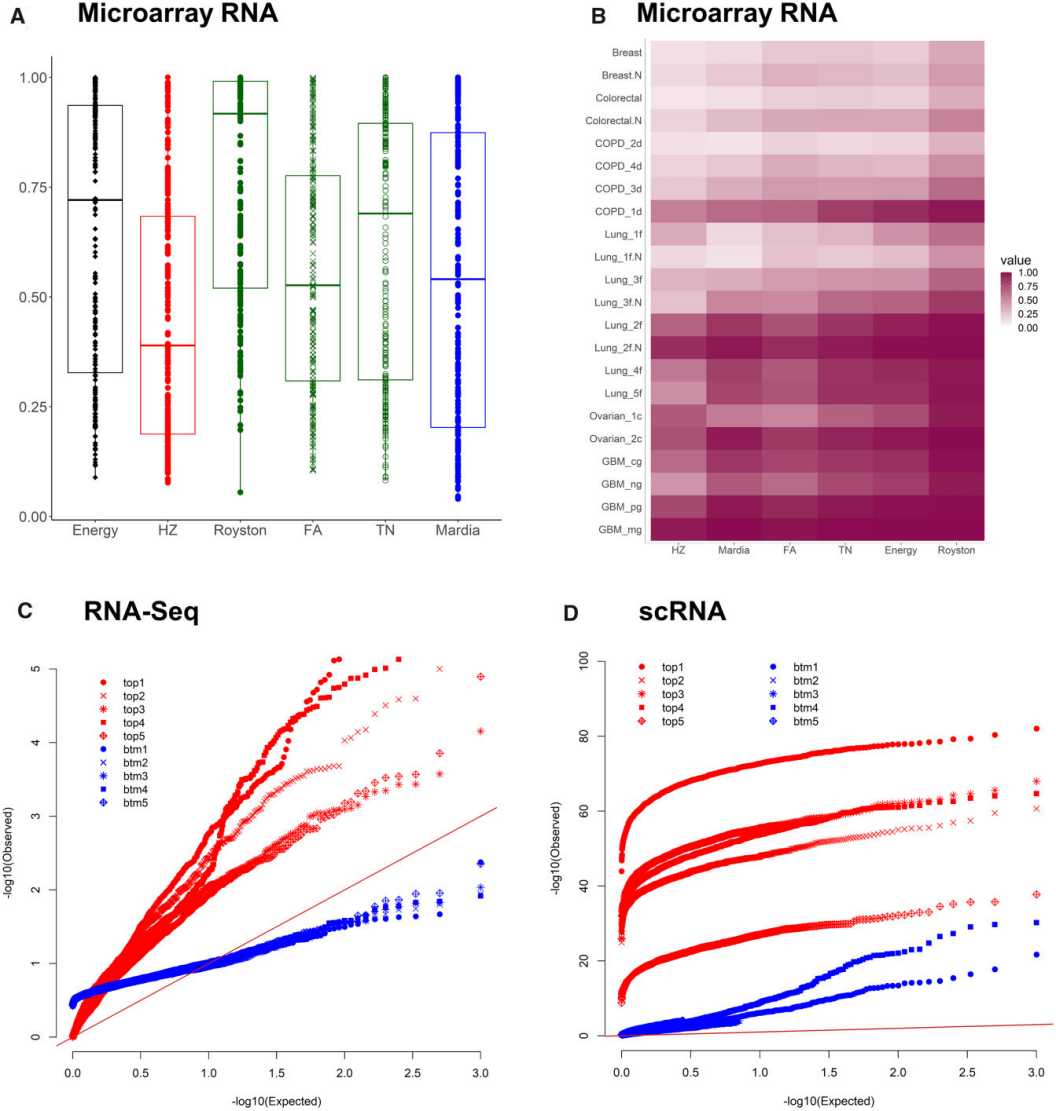
Results for the RNA empirical data under each of the six MVN tests. (A) Boxplots of the 220 rejection rates (Q's) per each MVN test for the microarray RNA data; 220 is the product of 22 (data sets) and 10 (pathways). (B) Heatmap of the average Q across pathways in (A). Each column in the heatmap contains 22 values. (C) The quantile–quantile plot of the *P*-values of the MVN tests for the RNA-seq data. The *y*-axis represents the transformed *P*-values derived from the test (observed) and the *x*-axis is the transformed theoretical quantile of *P*-values when the null hypothesis is true (expected). Red symbols are the *P*-values resulting in the top five leading rejection rates Q; while the blue symbols are *P*-values composing the bottom five Q. (D) The quantile–quantile plot of the *P*-values of the MVN tests for the scRNA-seq data from human heart left ventricle. Red symbols are the *P*-values composing the top five leading rejection rates Q; while the blue symbols are *P*-values composing the bottom five Q.

For each of the six MVN tests, the majority of the Q’s (across data sets and pathways/subclusters) are much greater than 5% as shown in the boxplots in Figure  1A when analyzing the microarray RNA values. This implies strong evidence of a non-MVN distribution. Details of these proportions are provided in the online supplementary materials (Supplementary Tables S1–S6). Another presentation in Figure  1B displays the average of the Q’s across the ten signaling pathways for each data set. The heatmap again demonstrates noticeable evidence against the normality assumption, as well as similarity in the rates of rejecting the MVN null hypothesis among the six tests. The corresponding average values are listed in the supplementary materials (Supplementary Table S7). For MVN tests with RNA-seq data in the 10 pathways, the top leading five and bottom five average Q are selected and the *P*-values generating the Q’s are presented in negative log-scale in a quantile–quantile plot in Figure  1C. The apparent deviation from the straight line in the plot strongly supports the existence of a non-MVN distribution for the RNA-seq values. For the scRNA-seq values of the human heart left ventricle, the same pattern is observed in Figure  1D for the subclusters. It is apparent that even the bottom five average Q’s (the five smallest rejection rates) are derived from very small *P*-values (blue symbols in Figure  1D), again implying significant and strong evidence of non-normality for the subclusters. The plots of the other three chambers demonstrate the same pattern and are displayed in the online supplementary material (Supplementary Figure S1).

**Figure 2:**
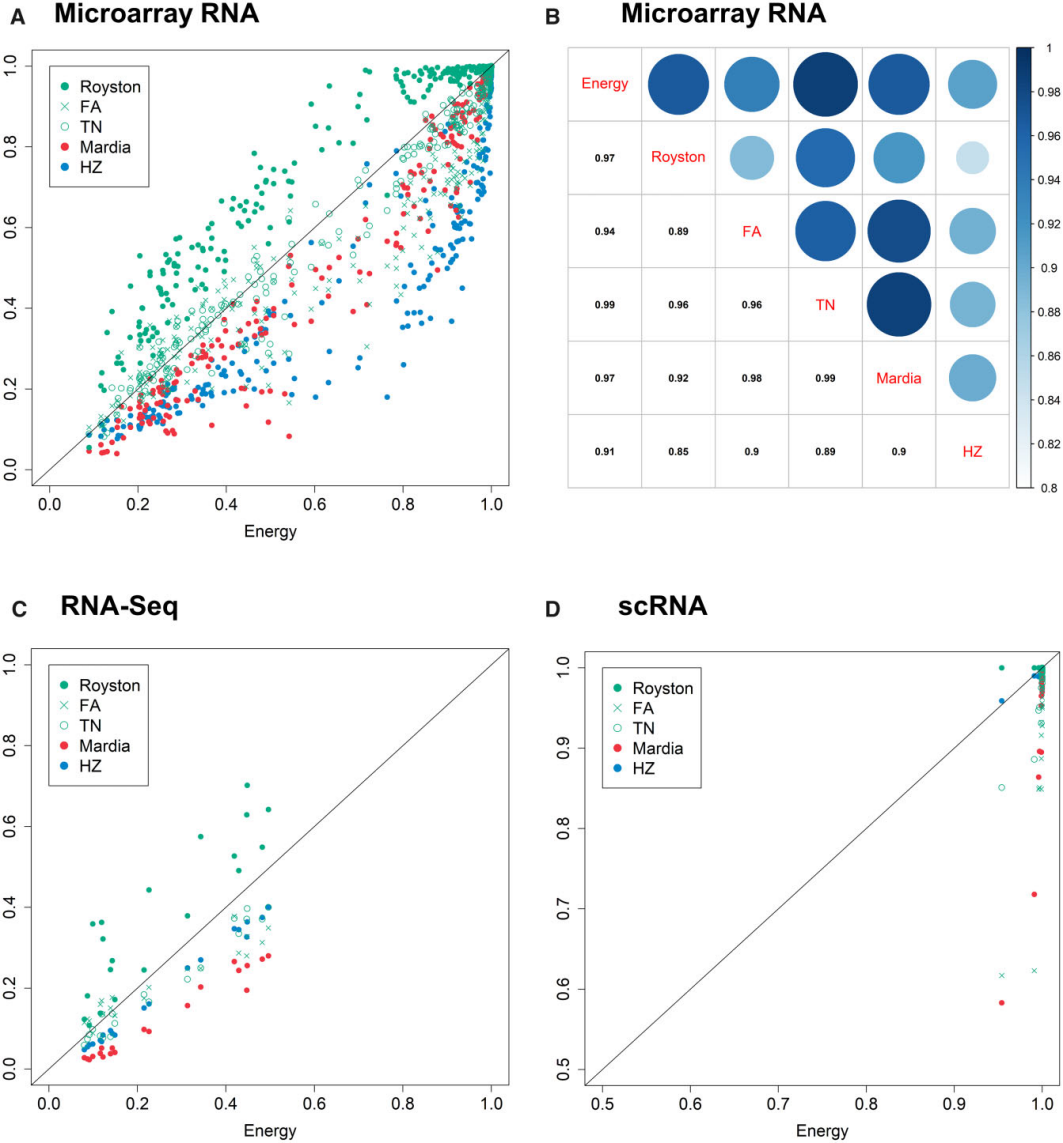
Rejection rates Q under different MVN tests when applied to different RNA empirical data. (A) The scatter plot of the rejection rates Q under one of the five MVN tests against the Q under the energy test when applied to the microarray RNA values. (B) The correlation of the rejection rates Q between any pair from the six MVN tests in (A). (C) The scatter plot of the rejection rates Q under one of the five MVN tests against the Q under the energy test when applied to the RNA-seq values. (D) The scatter plot of the rejection rates Q under one of the five MVN tests against the Q under the energy test when applied to the scRNA-seq data.

In addition, these six tests are fairly consistent with each other since their corresponding rejection rates (Q’s) are highly correlated. As indicated in the scatter plot in Figure  2A for the microarray RNA values, the Q’s show strong positive correlation, ranging between 0.85 and 0.90 as listed in the correlation plot in Figure  2B. When comparing the other MVN tests against the Energy test, the *x*-axis in Figure  2A, the scatter plot reveals that the Royston test tends to reject more often than the Energy test (indicated by points above the diagonal line); while both the HZ and Mardia tests are more conservative than the Energy test (points below the diagonal line). As for the FA and TN tests, their performance is relatively closer to that of the Energy test (points are close to the line). The pattern remains the same when investigating the RNA-seq data. In Figure  2C, the points contain the rejection rates from the tests on 10 pathways with data from cancer-free subjects and cancer patients, respectively. Though there are only 20 points in this subfigure, these rejection rates (Q’s) are still much larger than 5% and show the same positive correlation as in Figure  2A. For scRNA-seq, the results are demonstrated in Figure  2D. Since the *P*-values are very small, the corresponding rejection rates are all very close to 1, even those of the Mardia test are still much larger than 5%.

### Evaluating the impact of the distributional assumption

It has been demonstrated in previous sections that most gene expression values in gene-sets/pathways are not normally distributed, at least not in the RNA values from microarray, RNA-seq, and scRNA-seq applications we have tested. The next task was to examine if the non-normality affects the results from current gene set analysis methods. The following simulation studies are designed to mimic the case where the expression values are from a single-component distribution or from a mixture of more than one component distribution, for each of the two response groups termed as the case and control group. The distributions are all multi-dimensional containing weak and moderate correlation between gene expressions. The FCS GSAs are then performed and compared.

**Figure 3:**
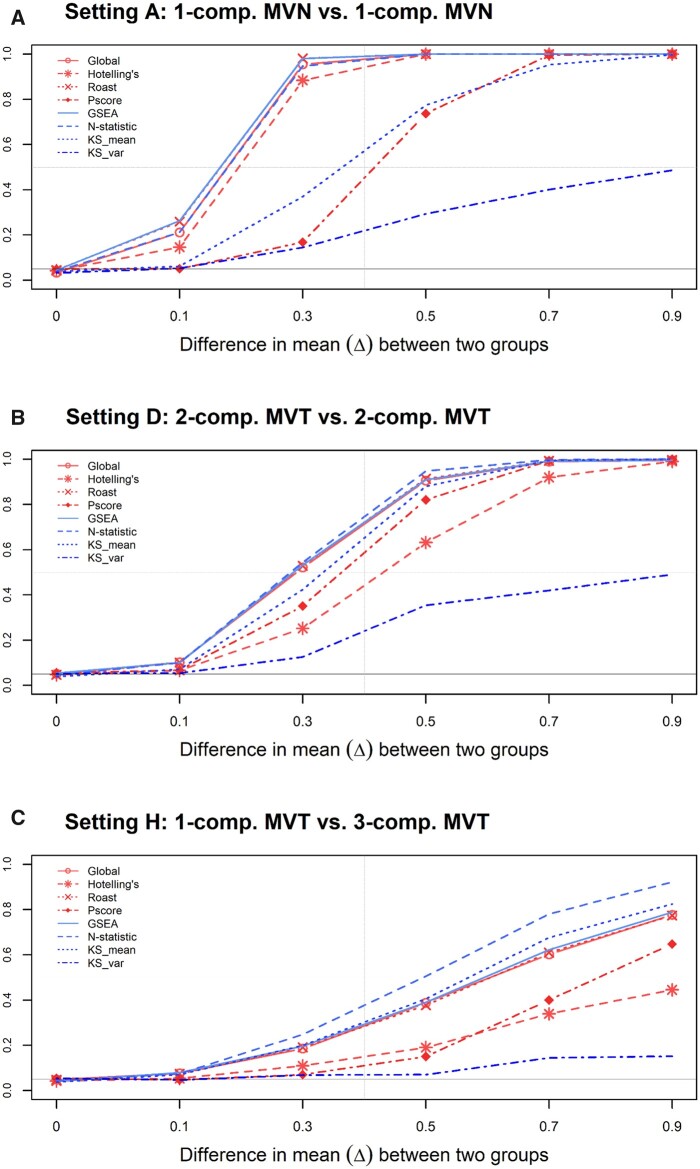
Power curves (when Δ > 0) of the eight GSA tools under selected simulation settings A, D, and H. When Δ= 0, the *y*-axis denotes the type I error rates. KS_mean and KS_var stand for the Kolmogrov–Smirnov test for the mean vector and for the covariance matrix, respectively. (A) Setting A, 1-component MVN *vs* 1-component MVN. (B) Setting D, 2-component mixture MVT *vs* 2-component mixture MVT. (C) Setting H, 1-component MVT *vs* 3-component mixture MVT.

In each of the three simulation settings, the difference in the mean of the two groups is denoted as $\underset{˜}{\Delta }=(\Delta ,\Delta ,\ldots ,\Delta ,)$, where Δ is set at 0 to evaluate the false positive rate (type I error rate) and set at 0.1, 0.3, 0.5, 0.7, and 0.9 to evaluate its power performance. A total of 1000 replications were carried out under each setting, where each replication contained 50 *control* subjects and 50 disease (*case*) subjects. Three selective results from setting A, D, and H are shown in Figure  3, A–C, respectively. The results of other settings are shown in the Supplementary Figure S2 and Tables S8–S15.

Generally, all tests maintain a reasonable type I error rate at the nominal level 0.05 (when Δ = 0), and their power curves rise as expected when the mean difference Δ increases. When comparing across the three subfigures, it is noticeable that the rate of increase of the power curves in setting A (Figure  3A) is fastest, followed by that in setting D (Figure  3B), and then setting H (Figure  3C). For instance, when Δ = 0.3, five out of the eight powers in Figure  3A are larger than 0.8; while all powers at Δ = 0.3 in Figure  3B are below 0.6 and below 0.4 in Figure  3C. This is not surprising because the simulated values in setting A are all from a MVN distribution, and the distance between the empirical distribution and MVN is the smallest, as measured by the KL divergence (Table  3). In contrast, the empirical distribution of the simulated values in the other two settings D and H is not that close to MVN.

The results from other settings reveal similar findings. For instance, the power performance under a single-component MVT distribution or a mixture of more than 1 MVT component, is always worse than that with MVN as the component distribution (Supplementary Figure S2). In addition, the pattern across settings A, C, and G, or across B, D, and F, remains the same as that in Figure  3.

When comparing these GSA tools in each subfigure in Figure  3 and in Supplementary Figure S2, we note that the global test and ROAST in the category of parametric GSAs and the GSEA and N-statistic in the nonparametric category perform better than the others. These four tools steadily outperform the rest, with the N-statistic standing out when the component distributions are MVT rather than MVN. In other words, the N-statistic is less sensitive to the normality assumption than the other GSA tools.

## Discussion

The findings in the first part of this study indicated strong evidence for rejecting the MVN distributional assumption for all three types of RNA values. The findings in the simulation studies in the second part further concluded that deviation from MVN can cause statistical power loss. This implies that using MVN simulated data may be a poor choice for benchmarking FCS tools. Among the GSA FCS tools examined, the nonparametric N-statistic performed comparatively well, at least in the limited scenarios investigated here. In other words, when RNA values come from subjects of different disease subtypes, the N-statistic is recommended for GSA.

However, there are limitations to this study. First, the simulations in the second part included either a single-component multivariate t-distribution or a mixture of distributions to denote the non-normally distributed populations. These choices may not represent well the true distributions of the biological data. Because the true distributions are usually unknown, the pattern of their non-normality can go beyond the cases considered here. In other words, unless all forms of non-normality can be defined and have undergone the same investigation as in this study, the results here cannot guarantee that the N-statistic will perform the best in those cases. A new GSA method that is robust to distributional assumptions remains in need of development. Until then, one solution in practice would be to try different GSAs and perform validation studies if there is consensus.

The second limitation to this study is the lack of measuring the degree of departure from normality, and hence this study did not identify a relationship between the degree of deviation and the loss of power of the GSAs. Traditional measurements include skewness, kurtosis, KL divergence, and Royston’s indices (Royston 1991), where each accounts for a type of non-normality. Future studies may focus on each of these and establish the trend of power loss in response to different degrees of departure. In this study, the mixture of distributions was adopted to represent the existence of sub-populations and it may not be proper to measure the skewness and kurtosis for these multi-mode distributions. The KL divergence between these mixture distributions and MVN, however, did not show a clear pattern. A more careful design of the simulated data would be needed in order to clarify the relationship between the relaxation of the distributional assumption and the performance of the FCS GSAs.

There are other issues in the development of GSAs requiring attention. First, since current statistical models may not describe properly the distribution of gene expression data under study (de Torrenté *et al.* 2019), a remedy for the simulation design may be to simulate from available databases where the sample size is big enough to cover the heterogeneity across individuals to serve as a pseudo-population. Similar ideas have been adopted by others (Lin *et al.* 2018). This solution may not be readily put into practice now, but it is not entirely infeasible. As long as data accumulation continues at a high speed and access remains open to users, such a choice should be available in the near future. Second, the GSA tools we consider here are limited to the FCS category. The GSAs in the over representation analysis category do not rely on the distributional assumption of the RNA values but rather on whether the genes are DE. Therefore, these GSAs are less affected by the MVN assumption and hence not discussed here. The third issue is, currently many new GSA methods are being developed based on given pathway topology (Draghici *et al.* 2007; Shojaie and Michailidis 2009; Chang *et al.* 2020). Most of these tools do not incorporate probabilistic randomness and statistical models, thus it is not easy to investigate their performance using statistical simulation studies to test their robustness against distributional assumptions. Effort would be required by the statistical community to formulate procedures for such evaluation. Finally, current GSA methods treat the gene-set or pathway as a deterministic group of genes. No uncertainty is considered, nor quantified. However, when the content of a pathway is retrieved from different pathway databases, it is quite possible that the derived pathways are different. Future development of GSA tools should incorporate this difference, either by taking the intersection or union of these component nodes, or by including a mechanism such as latent variables for this difference. More studies are clearly warranted.

## Data availability

All the human microarray RNA and RNA-seq gene expression data included in this study can be freely downloaded from the NCBI or TCGA website. GEO accession number or TCGA identification are listed in Table  1. The data management procedures and R code are documented in Supplementary File S2. The scRNA-seq expression values of the human heart samples can be downloaded from the Broad Institute Single Cell Portal (https://singlecell.broadinstitute.org/single_cell/study/SCP498/). The R code for the four MVN tests (Energy, Henze-Zirkler, Royston, and Mardia) can be downloaded from https://github.com/r05849032/Four_MVN_tests. For the parametric and nonparametric FCS GSA tools examined in this study, the software and functions are listed in Table  2.

Supplementary materials are available and include tables for MVN tests (Supplementary Tables S1–S6), average proportions across ten pathways for each data set and test (Supplementary Table S7), and for GSA evaluation (Supplementary Tables S8–S15). Supplementary File S1 contains background information on the six MVN tests. Supplementary File S2 contains the R code for data management and normalization. The R code for the four MVN tests can be downloaded from https://github.com/r05849032/Four_MVN_tests.

Supplementary material is available at *G3* online.

## Supplementary Material

jkab365_Supplementary_Figures

jkab365_Supplementary_Tables

jkab365_Supplementary_FileS1

jkab365_Supplementary_FileS2
