# Supplementary figures and images for: The misuse of distributional assumptions in functional class scoring gene-set and pathway analysis
Source: G3 (Bethesda). 2021 Oct 25;12(1):jkab365. doi: 10.1093/g3journal/jkab365 (PMC8728032; doi:10.1093/g3journal/jkab365)

(a) Right Atrium (RA)

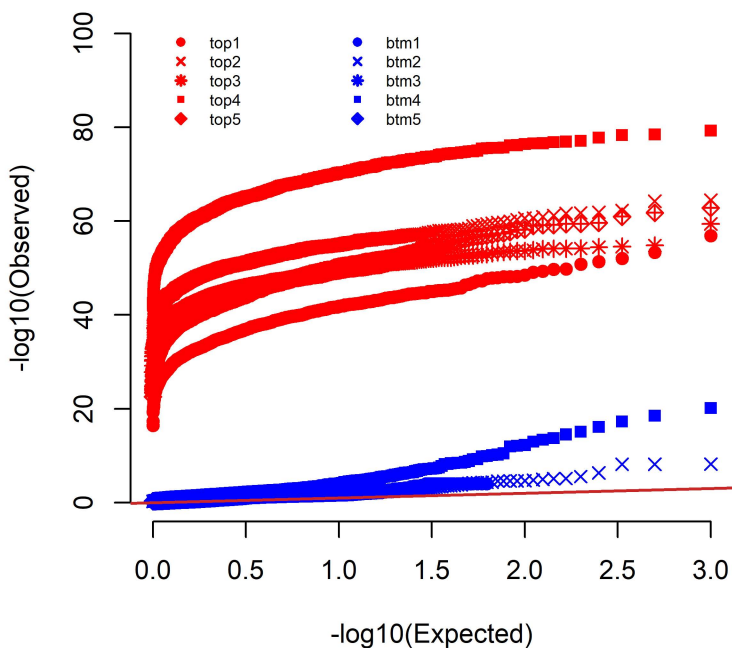

(b) Right Ventricle (RV)

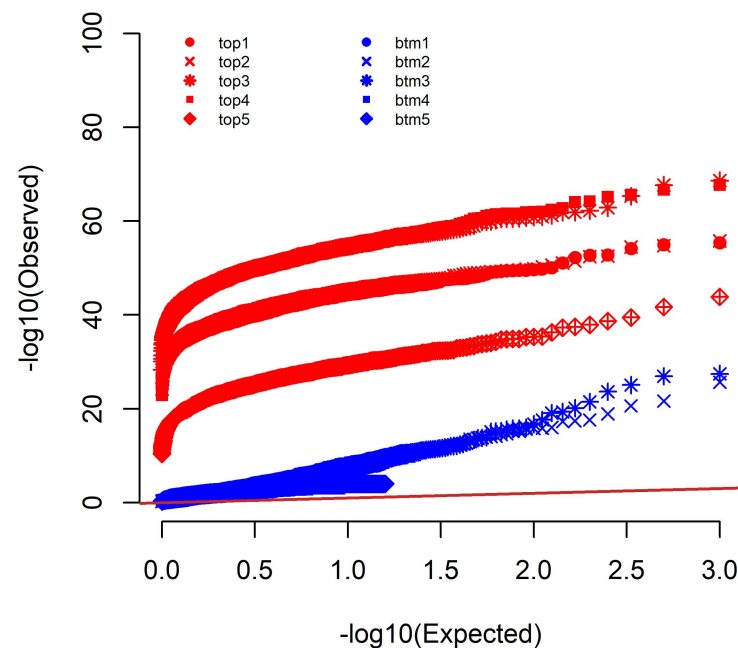

(c) Left Atrium (LA)

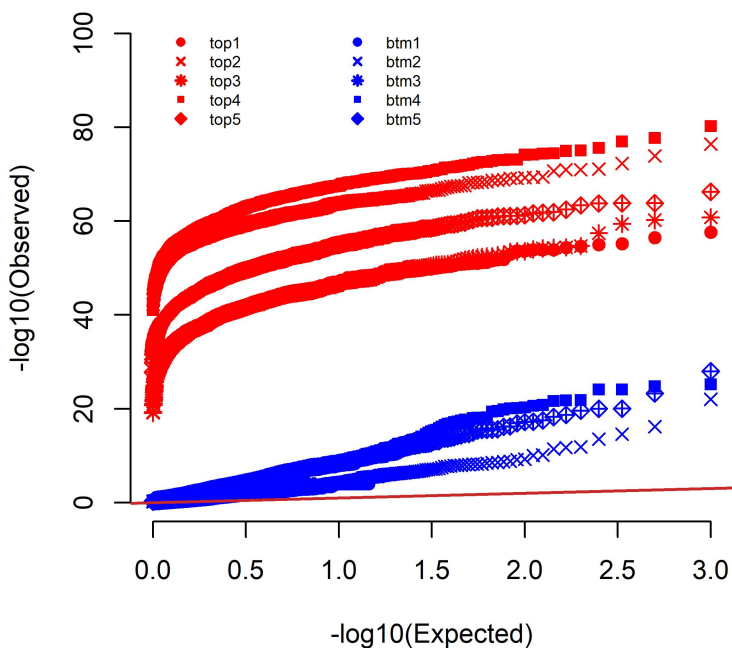

(d) Left Ventricle (LV)

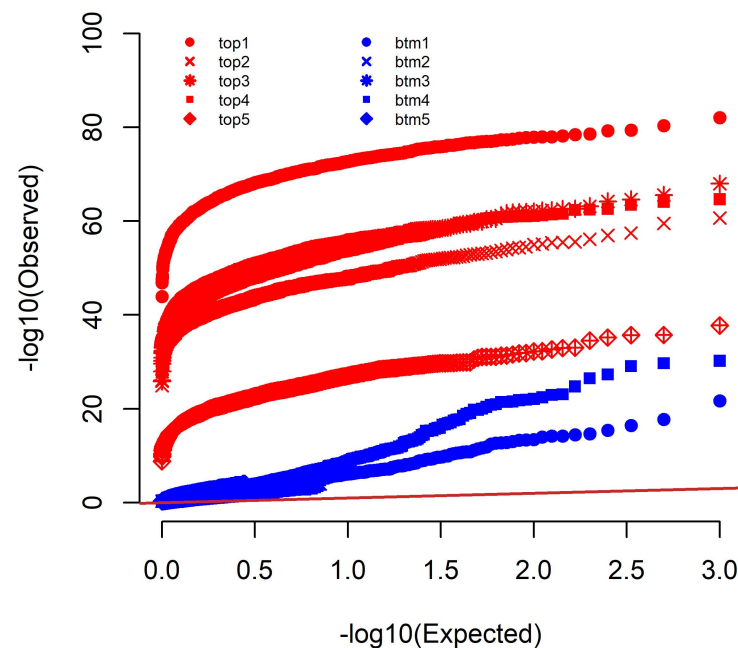

Figure S2

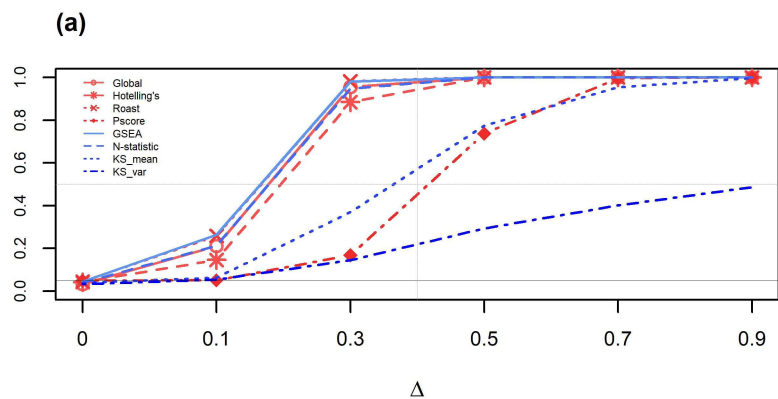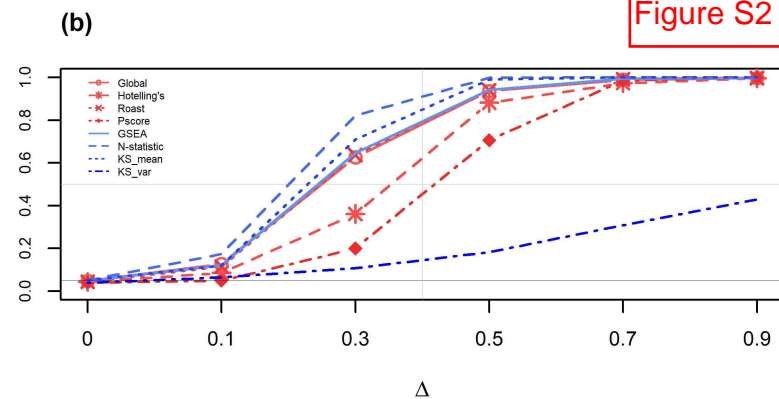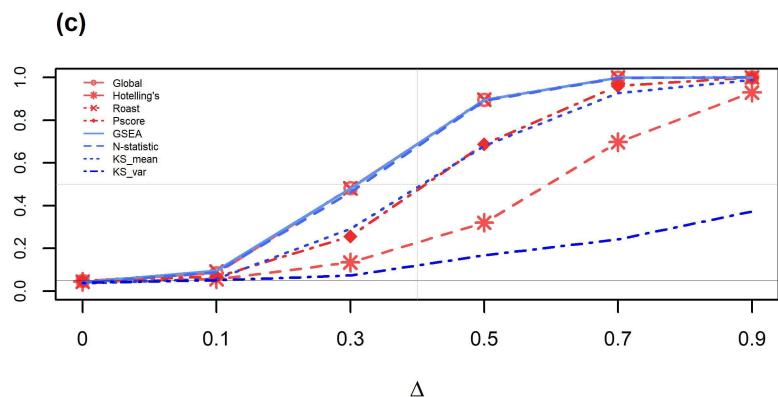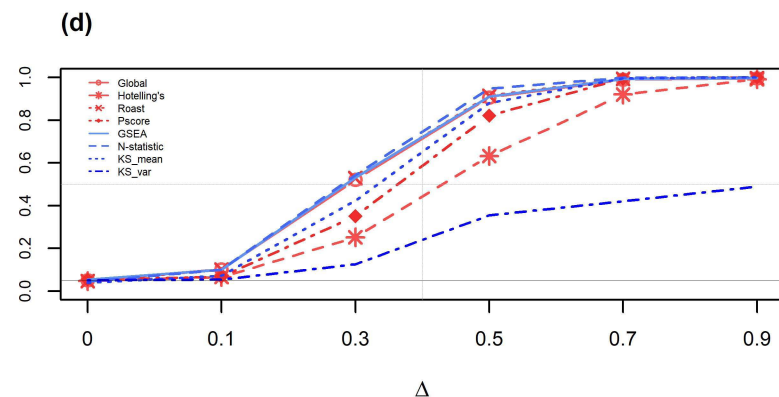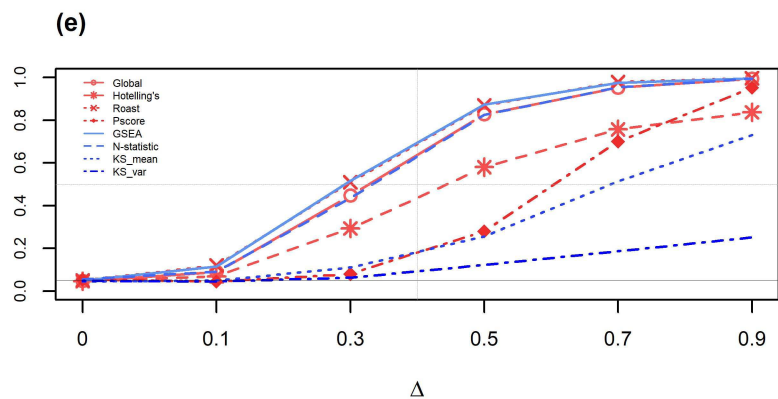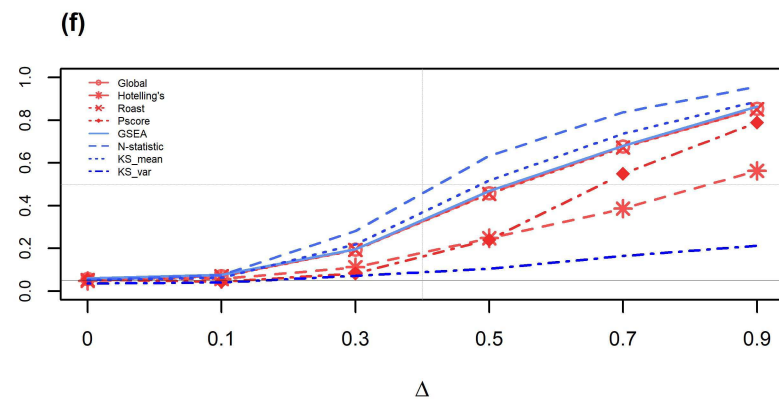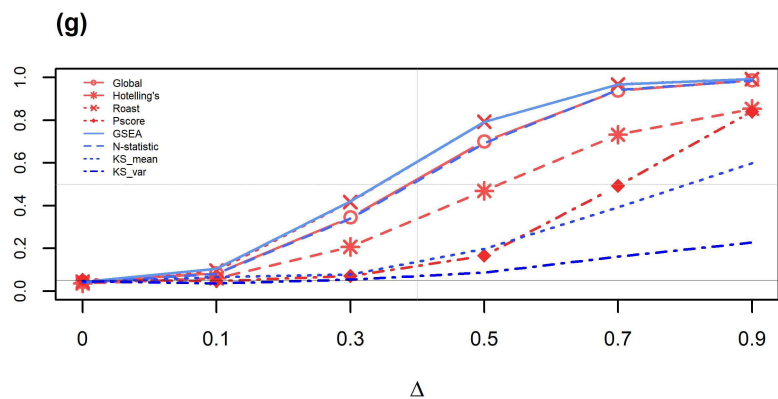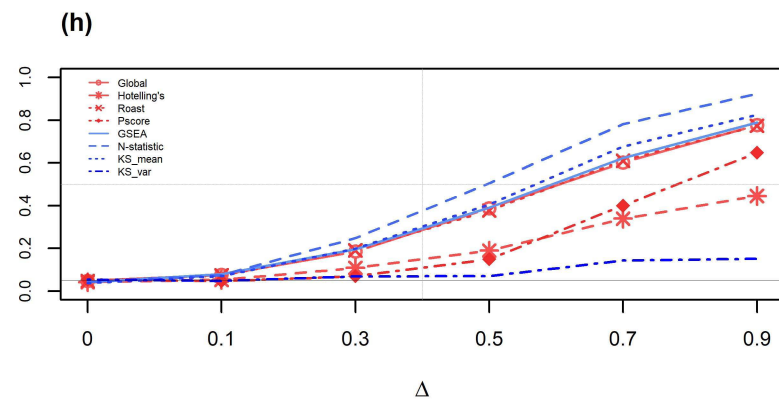

Supplement: jkab365_Supplementary_Figures [file jkab365_supplementary_figures.pdf]
