# Supplementary material for: The misuse of distributional assumptions in functional class scoring gene-set and pathway analysis
Source: G3 (Bethesda). 2021 Oct 25;12(1):jkab365. doi: 10.1093/g3journal/jkab365 (PMC8728032; doi:10.1093/g3journal/jkab365)
Supplement: jkab365_Supplementary_Tables [file jkab365_supplementary_tables.pdf]

Table S1. Energy Test: These are the proportions Q, $Q = \frac{\#\{p_i \le 0.05\}}{1000}$, of p values less than the nominal 0.05 among 1000 replications with respect to each data set (row) and pathway (column).

	P53	mTOR	Jak-STAT	PI3k-Akt	Wnt	ERBB	MAPK	RAS	TGF_BETA	TNF
Breast	0.216	0.162	0.247	0.155	0.322	0.256	0.219	0.209	0.118	0.290
Colorectal	0.203	0.205	0.204	0.204	0.224	0.227	0.153	0.243	0.250	0.167
Lung_1f	0.687	0.367	0.282	0.480	0.532	0.542	0.499	0.495	0.445	0.441
Lung_2f	0.981	0.934	0.940	0.960	0.978	0.986	0.928	0.966	0.939	0.861
Lung_3f	0.359	0.427	0.489	0.464	0.507	0.477	0.413	0.469	0.655	0.480
Lung_4f	0.929	0.923	0.915	0.969	0.917	0.978	0.837	0.799	0.950	0.922
Lung_5f	0.825	0.913	0.912	0.891	0.900	0.910	0.810	0.934	0.935	0.908
Ovarian_1c	0.795	0.718	0.715	0.785	0.895	0.870	0.784	0.592	0.902	0.724
Ovarian_2c	0.924	0.951	0.982	0.969	0.981	0.995	0.981	0.983	0.994	0.987
GBM_cg	0.974	0.865	0.851	0.860	0.867	0.944	0.885	0.873	0.917	0.973
GBM_mg	1.000	1.000	1.000	1.000	0.999	1.000	0.999	0.999	1.000	0.997
GBM_ng	0.910	0.842	0.955	0.825	0.817	0.862	0.803	0.811	0.847	0.785
GBM_pg	0.999	0.992	1.000	0.987	0.987	0.971	0.981	0.997	0.995	0.978
Breast.N	0.233	0.320	0.141	0.347	0.336	0.345	0.290	0.206	0.398	0.234
Colorectal.N	0.311	0.367	0.541	0.346	0.329	0.463	0.353	0.450	0.235	0.365
Lung_1f.N	0.286	0.264	0.271	0.263	0.267	0.434	0.278	0.336	0.267	0.152
Lung_2f.N	0.992	0.989	0.990	0.997	0.998	0.999	0.996	0.998	0.997	0.973
Lung_3f.N	0.633	0.602	0.932	0.698	0.548	0.764	0.631	0.801	0.702	0.554
COPD_1d	0.857	0.952	0.920	0.921	0.878	0.839	0.908	0.922	0.968	0.926
COPD_2d	0.227	0.257	0.089	0.116	0.182	0.130	0.210	0.123	0.267	0.253
COPD_3d	0.353	0.507	0.395	0.431	0.389	0.416	0.490	0.294	0.468	0.616
COPD_4d	0.256	0.395	0.183	0.251	0.287	0.202	0.381	0.545	0.282	0.286

Table S2. HZ Test: These are the proportions Q, $Q = \frac{\#\{p_i \le 0.05\}}{1000}$, of p values less than the nominal 0.05 among 1000 replications with respect to each data set (row) and pathway (column).

	P53	mTOR	Jak-STAT	PI3k-Akt	Wnt	ERBB	MAPK	RAS	TGF_BETA	TNF
Breast	0.144	0.084	0.135	0.085	0.175	0.158	0.130	0.118	0.112	0.172
Colorectal	0.104	0.129	0.103	0.101	0.152	0.130	0.077	0.118	0.128	0.107
Lung_1f	0.554	0.272	0.206	0.350	0.417	0.505	0.384	0.393	0.323	0.293
Lung_2f	0.787	0.640	0.616	0.753	0.744	0.768	0.598	0.711	0.738	0.481
Lung_3f	0.211	0.308	0.347	0.329	0.387	0.338	0.264	0.268	0.468	0.349
Lung_4f	0.654	0.638	0.611	0.712	0.601	0.705	0.450	0.353	0.610	0.599
Lung_5f	0.389	0.549	0.521	0.531	0.499	0.368	0.388	0.450	0.571	0.557
Ovarian_1c	0.678	0.758	0.661	0.790	0.838	0.696	0.734	0.563	0.856	0.706
Ovarian_2c	0.650	0.639	0.817	0.670	0.817	0.887	0.721	0.689	0.895	0.859
GBM_cg	0.847	0.512	0.667	0.635	0.605	0.762	0.545	0.548	0.652	0.774
GBM_mg	0.988	0.981	1.000	0.986	0.954	0.979	0.940	0.958	0.974	0.875
GBM_ng	0.674	0.363	0.572	0.489	0.507	0.375	0.454	0.356	0.454	0.418
GBM_pg	0.981	0.792	0.946	0.775	0.795	0.690	0.700	0.735	0.854	0.682
Breast.N	0.129	0.183	0.099	0.199	0.204	0.188	0.140	0.107	0.232	0.134
Colorectal.N	0.171	0.183	0.274	0.159	0.196	0.200	0.188	0.222	0.147	0.191
Lung_1f.N	0.157	0.157	0.154	0.184	0.192	0.237	0.200	0.172	0.160	0.123
Lung_2f.N	0.846	0.904	0.901	0.934	0.946	0.968	0.924	0.977	0.897	0.781
Lung_3f.N	0.293	0.180	0.611	0.253	0.186	0.180	0.216	0.260	0.277	0.226
COPD_1d	0.471	0.609	0.530	0.605	0.545	0.390	0.514	0.565	0.748	0.567
COPD_2d	0.122	0.168	0.086	0.083	0.111	0.122	0.135	0.099	0.212	0.197
COPD_3d	0.210	0.264	0.191	0.243	0.236	0.195	0.245	0.141	0.313	0.414
COPD_4d	0.114	0.245	0.134	0.161	0.172	0.109	0.254	0.382	0.200	0.174

Table S3. Royston Test: These are the proportions Q, $Q = \frac{\#\{p_i \le 0.05\}}{1000}$, of p values less than the nominal 0.05 among 1000 replications with respect to each data set (row) and pathway (column).

	P53	mTOR	Jak-STAT	PI3k-Akt	Wnt	ERBB	MAPK	RAS	TGF_BETA	TNF
Breast	0.338	0.264	0.451	0.279	0.551	0.405	0.347	0.401	0.240	0.535
Colorectal	0.366	0.264	0.530	0.334	0.320	0.490	0.264	0.340	0.449	0.248
Lung_1f	0.809	0.574	0.511	0.649	0.740	0.614	0.600	0.560	0.664	0.642
Lung_2f	0.998	0.987	0.990	0.998	0.993	0.997	0.977	0.984	0.980	0.975
Lung_3f	0.598	0.611	0.713	0.639	0.709	0.749	0.646	0.701	0.810	0.685
Lung_4f	0.953	0.984	0.963	0.992	0.984	0.996	0.965	0.951	0.973	0.991
Lung_5f	0.975	0.981	0.993	0.982	0.984	0.985	0.923	0.985	0.995	0.963
Ovarian_1c	0.961	0.867	0.990	0.969	0.987	0.981	0.976	0.906	0.994	0.986
Ovarian_2c	0.998	0.999	1.000	0.996	1.000	1.000	0.998	0.999	1.000	0.999
GBM_cg	0.995	0.992	0.996	0.988	0.983	0.998	0.971	0.968	0.990	1.000
GBM_mg	1.000	1.000	1.000	1.000	1.000	1.000	0.999	0.999	1.000	0.998
GBM_ng	0.984	0.981	0.99	0.911	0.912	0.952	0.982	0.984	0.924	0.991
GBM_pg	1.000	0.996	1.000	0.996	0.982	0.993	0.991	0.993	0.991	0.998
Breast.N	0.330	0.470	0.392	0.543	0.502	0.459	0.504	0.320	0.519	0.283
Colorectal.N	0.374	0.610	0.715	0.548	0.446	0.784	0.448	0.617	0.351	0.548
Lung_1f.N	0.572	0.437	0.520	0.533	0.364	0.603	0.409	0.557	0.514	0.264
Lung_2f.N	0.998	0.995	0.997	1.000	0.999	1.000	0.997	0.997	1.000	0.986
Lung_3f.N	0.951	0.851	0.984	0.900	0.713	0.680	0.847	0.936	0.930	0.760
COPD_1d	0.941	0.957	0.994	0.976	0.997	0.988	0.968	0.963	0.991	0.972
COPD_2d	0.481	0.486	0.055	0.208	0.328	0.340	0.400	0.197	0.421	0.435
COPD_3d	0.495	0.744	0.541	0.654	0.666	0.672	0.717	0.575	0.658	0.793
COPD_4d	0.494	0.491	0.455	0.443	0.487	0.355	0.521	0.678	0.505	0.456

Table S4. Mardia Test: These are the proportions Q, $Q = \frac{\#\{p_i \leq 0.05\}}{1000}$, of p values less than the nominal 0.05 among 1000 replications with respect to each data set (row) and pathway (column).

	P53	mTOR	Jak-STAT	PI3k-Akt	Wnt	ERBB	MAPK	RAS	TGF_BETA	TNF
Breast	0.147	0.156	0.203	0.121	0.263	0.187	0.160	0.115	0.042	0.172
Colorectal	0.128	0.137	0.155	0.169	0.119	0.114	0.105	0.164	0.193	0.079
Lung_1f	0.392	0.110	0.089	0.193	0.188	0.083	0.195	0.118	0.158	0.201
Lung_2f	0.924	0.874	0.862	0.891	0.942	0.929	0.833	0.867	0.867	0.715
Lung_3f	0.256	0.311	0.339	0.332	0.382	0.349	0.303	0.417	0.526	0.334
Lung_4f	0.770	0.803	0.853	0.931	0.808	0.953	0.754	0.706	0.885	0.884
Lung_5f	0.653	0.812	0.875	0.825	0.811	0.824	0.698	0.884	0.904	0.846
Ovarian_1c	0.550	0.409	0.620	0.561	0.693	0.739	0.556	0.368	0.756	0.486
Ovarian_2c	0.906	0.947	0.970	0.956	0.942	0.996	0.965	0.974	0.988	0.989
GBM_cg	0.956	0.876	0.866	0.796	0.854	0.911	0.831	0.843	0.906	0.917
GBM_mg	1.000	0.999	1.000	1.000	1.000	0.999	0.999	0.997	1.000	0.995
GBM_ng	0.827	0.736	0.825	0.733	0.738	0.818	0.699	0.682	0.693	0.586
GBM_pg	0.991	0.977	1.000	0.979	0.976	0.931	0.946	0.983	0.984	0.957
Breast.N	0.188	0.283	0.068	0.309	0.277	0.278	0.237	0.185	0.344	0.196
Colorectal.N	0.171	0.307	0.504	0.293	0.195	0.398	0.286	0.362	0.128	0.274
Lung_1f.N	0.173	0.135	0.131	0.096	0.091	0.202	0.097	0.174	0.159	0.040
Lung_2f.N	0.976	0.963	0.960	0.976	0.990	0.998	0.967	0.990	0.987	0.874
Lung_3f.N	0.501	0.496	0.800	0.572	0.458	0.566	0.430	0.634	0.490	0.360
COPD_1d	0.591	0.712	0.643	0.687	0.495	0.622	0.654	0.696	0.817	0.626
COPD_2d	0.225	0.120	0.046	0.062	0.151	0.045	0.131	0.043	0.198	0.221
COPD_3d	0.280	0.399	0.372	0.344	0.307	0.411	0.375	0.240	0.368	0.475
COPD_4d	0.205	0.277	0.105	0.167	0.248	0.156	0.322	0.531	0.215	0.218

Table S5. FA Test: These are the proportions Q, $Q = \frac{\#\{p_i \leq 0.05\}}{1000}$, of p values less than the nominal 0.05 among 1000 replications with respect to each data set (row) and pathway (column).

	P53	mTOR	Jak-STAT	PI3k-Akt	Wnt	ERBB	MAPK	RAS	TGF_BETA	TNF
Breast	0.236	0.290	0.280	0.159	0.387	0.279	0.212	0.181	0.108	0.278
Colorectal	0.212	0.217	0.226	0.242	0.189	0.189	0.172	0.250	0.297	0.121
Lung_1f	0.506	0.201	0.280	0.252	0.291	0.166	0.281	0.209	0.206	0.310
Lung_2f	0.793	0.729	0.722	0.813	0.832	0.867	0.710	0.790	0.764	0.527
Lung_3f	0.297	0.347	0.399	0.387	0.466	0.504	0.398	0.511	0.572	0.368
Lung_4f	0.647	0.713	0.725	0.772	0.789	0.906	0.623	0.543	0.790	0.774
Lung_5f	0.581	0.747	0.858	0.753	0.718	0.753	0.635	0.834	0.852	0.741
Ovarian_1c	0.434	0.305	0.653	0.499	0.682	0.683	0.525	0.398	0.709	0.410
Ovarian_2c	0.738	0.774	0.924	0.808	0.868	0.964	0.765	0.886	0.973	0.891
GBM_cg	0.888	0.855	0.867	0.718	0.782	0.888	0.685	0.769	0.866	0.794
GBM_mg	0.992	0.987	0.999	0.995	0.974	0.989	0.967	0.964	0.997	0.955
GBM_ng	0.719	0.551	0.735	0.660	0.711	0.623	0.656	0.671	0.725	0.455
GBM_pg	0.979	0.913	0.986	0.961	0.918	0.859	0.834	0.928	0.957	0.887
Breast.N	0.303	0.350	0.158	0.410	0.452	0.330	0.360	0.311	0.454	0.276
Colorectal.N	0.217	0.399	0.526	0.405	0.246	0.551	0.420	0.467	0.181	0.321
Lung_1f.N	0.321	0.249	0.369	0.229	0.204	0.305	0.236	0.333	0.254	0.138
Lung_2f.N	0.918	0.909	0.899	0.942	0.964	0.942	0.898	0.939	0.952	0.741
Lung_3f.N	0.469	0.449	0.626	0.574	0.471	0.505	0.446	0.599	0.468	0.438
COPD_1d	0.641	0.704	0.696	0.727	0.539	0.669	0.657	0.707	0.807	0.656
COPD_2d	0.358	0.191	0.105	0.132	0.183	0.128	0.166	0.107	0.298	0.288
COPD_3d	0.357	0.523	0.468	0.411	0.397	0.471	0.389	0.296	0.500	0.560
COPD_4d	0.321	0.422	0.228	0.288	0.378	0.304	0.443	0.642	0.329	0.354

Table S6. TN Test: These are the proportions Q, $Q = \frac{\#\{p_i \le 0.05\}}{1000}$, of p values less than the nominal 0.05 among 1000 replications with respect to each data set (row) and pathway (column).

	P53	mTOR	Jak-STAT	PI3k-Akt	Wnt	ERBB	MAPK	RAS	TGF_BETA	TNF
Breast	0.195	0.202	0.317	0.177	0.393	0.277	0.238	0.220	0.117	0.320
Colorectal	0.226	0.177	0.303	0.230	0.231	0.228	0.161	0.261	0.270	0.157
Lung_1f	0.532	0.239	0.251	0.324	0.379	0.287	0.311	0.289	0.298	0.311
Lung_2f	0.922	0.889	0.875	0.902	0.937	0.938	0.843	0.883	0.837	0.752
Lung_3f	0.359	0.410	0.453	0.459	0.479	0.491	0.410	0.529	0.590	0.473
Lung_4f	0.841	0.894	0.892	0.947	0.865	0.981	0.801	0.772	0.932	0.891
Lung_5f	0.799	0.874	0.913	0.903	0.884	0.915	0.800	0.933	0.947	0.918
Ovarian_1c	0.715	0.582	0.742	0.697	0.804	0.812	0.683	0.502	0.805	0.604
Ovarian_2c	0.848	0.908	0.975	0.925	0.931	0.974	0.941	0.947	0.961	0.945
GBM_cg	0.940	0.849	0.881	0.802	0.843	0.896	0.850	0.832	0.873	0.929
GBM_mg	1.000	1.000	1.000	1.000	0.999	0.998	0.993	0.995	1.000	0.985
GBM_ng	0.864	0.803	0.895	0.782	0.727	0.844	0.799	0.779	0.742	0.708
GBM_pg	0.991	0.974	1.000	0.980	0.976	0.923	0.942	0.985	0.976	0.945
Breast.N	0.236	0.324	0.203	0.373	0.345	0.358	0.368	0.226	0.411	0.237
Colorectal.N	0.248	0.397	0.622	0.395	0.282	0.519	0.347	0.478	0.221	0.372
Lung_1f.N	0.313	0.245	0.230	0.212	0.191	0.311	0.200	0.293	0.227	0.136
Lung_2f.N	0.959	0.934	0.963	0.978	0.993	0.990	0.956	0.984	0.990	0.887
Lung_3f.N	0.634	0.658	0.858	0.718	0.559	0.642	0.556	0.769	0.704	0.490
COPD_1d	0.795	0.853	0.853	0.855	0.798	0.807	0.851	0.847	0.950	0.853
COPD_2d	0.255	0.211	0.089	0.112	0.196	0.091	0.184	0.082	0.250	0.264
COPD_3d	0.342	0.466	0.405	0.434	0.408	0.416	0.438	0.315	0.424	0.563
COPD_4d	0.295	0.363	0.202	0.285	0.355	0.200	0.383	0.513	0.304	0.309

Table S7: Average proportions Q across ten signaling pathways for each dataset with respect to each of the MVN test.

	Energy	HZ	Royston	FA	TN	Mardia
Breast	0.2194	0.1313	0.3811	0.241	0.2456	0.1566
Colorectal	0.208	0.1149	0.3605	0.2115	0.2244	0.1363
Lung_1f	0.477	0.3697	0.6363	0.2702	0.3221	0.1727
Lung_2f	0.9473	0.6836	0.9879	0.7547	0.8778	0.8704
Lung_3f	0.474	0.3269	0.6861	0.4249	0.4653	0.3549
Lung_4f	0.9139	0.5933	0.9752	0.7282	0.8816	0.8347
Lung_5f	0.8938	0.4823	0.9766	0.7472	0.8886	0.8132
Ovarian_1c	0.778	0.728	0.9617	0.5298	0.6946	0.5738
Ovarian_2c	0.9747	0.7644	0.9989	0.8591	0.9355	0.9633
GBM_cg	0.9009	0.6547	0.9881	0.8112	0.8695	0.8756
GBM_mg	0.9994	0.9635	0.9996	0.9819	0.997	0.9989
GBM_ng	0.8457	0.4662	0.9611	0.6506	0.7943	0.7337
GBM_pg	0.9887	0.795	0.994	0.9222	0.9692	0.9724
Breast.N	0.285	0.1615	0.4322	0.3404	0.3081	0.2365
Colorectal.N	0.376	0.1931	0.5441	0.3733	0.3881	0.2918
Lung_1f.N	0.2818	0.1736	0.4773	0.2638	0.2358	0.1298
Lung_2f.N	0.9929	0.9078	0.9969	0.9104	0.9634	0.9681
Lung_3f.N	0.6865	0.2682	0.8552	0.5045	0.6588	0.5307
COPD_1d	0.9091	0.5544	0.9747	0.6803	0.8462	0.6543
COPD_2d	0.1854	0.1335	0.3351	0.1956	0.1734	0.1242
COPD_3d	0.4359	0.2452	0.6515	0.4372	0.4211	0.3571
COPD_4d	0.3068	0.1945	0.4885	0.3709	0.3209	0.2444

Table S8: Proportion of rejection for each of the GSAs under the following distributions

Control group	Case group
MVN($\underset{\sim}{\mathbf{0}}_{30\times1}, \mathbf{\Sigma}_1$)	MVN($\underset{\sim}{\mathbf{\Delta}}_{30\times1}, \mathbf{\Sigma}_1$)

Δ	Parametric				Non-parametric			
	Global	Hotelling	Roast	P score	GSEA	N-Statistic	KS_mean	KS_var
0	0.033	0.042	0.045	0.049	0.044	0.039	0.038	0.032
0.1	0.211	0.146	0.259	0.051	0.263	0.212	0.062	0.053
0.3	0.955	0.884	0.981	0.168	0.981	0.947	0.370	0.145
0.5	1	1	1	0.737	1	1	0.774	0.293
0.7	1	1	1	0.996	1	1	0.953	0.401
0.9	1	1	1	1	1	1	0.997	0.486

Table S9: Proportion of rejection for each of the GSAs under the following distributions

Control group	Case group
MVT($\underset{\sim}{\mathbf{0}}_{30\times1}, \mathbf{\Sigma}_2$)	MVT($\underset{\sim}{\mathbf{\Delta}}_{30\times1}, \mathbf{\Sigma}_2$)

Δ	Parametric				Non-parametric			
	Global	Hotelling	Roast	P score	GSEA	N-Statistic	KS_mean	KS_var
0	0.047	0.043	0.044	0.040	0.047	0.051	0.051	0.038
0.1	0.127	0.085	0.115	0.049	0.121	0.173	0.119	0.064
0.3	0.626	0.361	0.637	0.200	0.646	0.822	0.709	0.108
0.5	0.936	0.881	0.940	0.706	0.941	0.999	0.990	0.183
0.7	0.989	0.973	0.992	0.985	0.994	1	1	0.308
0.9	0.997	0.996	0.998	1	0.998	1	1	0.430

Table S10: Proportion of rejection for each of the GSAs under the following distributions

Control group	Case group
$.5\text{MVN}(\underset{\sim}{\mathbf{0}}_{30\times1}, \mathbf{\Sigma}_1) + .5\text{MVN}(\underset{\sim}{\mathbf{1}}_{30\times1}, \mathbf{\Sigma}_2)$	$0.5\text{MVN}(\underset{\sim}{\mathbf{0}}_{30\times1} + \underset{\sim}{\mathbf{\Delta}}_{30\times1}, \mathbf{\Sigma}_1) + 0.5\text{MVN}(\underset{\sim}{\mathbf{1}} + \underset{\sim}{\mathbf{\Delta}}, \mathbf{\Sigma}_2)$

Δ	Parametric				Non-parametric			
	Global	Hotelling	Roast	P score	GSEA	N-Statistic	KS_mean	KS_var
0	0.047	0.044	0.044	0.047	0.042	0.039	0.036	0.039
0.1	0.084	0.056	0.092	0.067	0.095	0.088	0.057	0.050
0.3	0.482	0.134	0.481	0.256	0.480	0.462	0.292	0.073
0.5	0.895	0.320	0.896	0.687	0.895	0.890	0.679	0.168
0.7	0.999	0.697	0.999	0.960	0.999	0.997	0.927	0.242
0.9	1	0.929	1	1	1	1	0.988	0.372

Table S11: Proportion of rejection for each of the GSAs under the following distributions

Control group	Case group
$.5\text{MVT}(\underset{\sim}{\mathbf{0}}_{30\times1}, \mathbf{\Sigma}_1) + .5\text{MVT}(\underset{\sim}{\mathbf{1}}_{30\times1}, \mathbf{\Sigma}_2)$	$0.5\text{MVT}(\underset{\sim}{\mathbf{0}}_{30\times1} + \underset{\sim}{\mathbf{\Delta}}_{30\times1}, \mathbf{\Sigma}_1) + 0.5\text{MVT}(\underset{\sim}{\mathbf{1}}_{30\times1} + \underset{\sim}{\mathbf{\Delta}}_{30\times1}, \mathbf{\Sigma}_2)$

Δ	Parametric				Non-parametric			
	Global	Hotelling	Roast	P score	GSEA	N-Statistic	KS_mean	KS_var
0	0.051	0.047	0.047	0.054	0.054	0.045	0.038	0.051
0.1	0.102	0.066	0.101	0.067	0.100	0.100	0.071	0.054
0.3	0.521	0.252	0.529	0.351	0.532	0.544	0.424	0.125
0.5	0.906	0.632	0.914	0.821	0.910	0.948	0.880	0.354
0.7	0.990	0.920	0.993	0.994	0.992	0.998	0.995	0.420
0.9	0.996	0.991	0.997	1	0.997	1	1	0.490

Table S12: Proportion of rejection for each of the GSAs under the following distributions

Control group	Case group
MVN($\underset{\sim}{\mathbf{0}}_{30\times1}, \mathbf{\Sigma}_1$)	0.5MVN($\underset{\sim}{\mathbf{0}}_{30\times1}, \mathbf{\Sigma}_1$) + 0.5MVN($\underset{\sim}{\mathbf{\Delta}}_{30\times1}, \mathbf{\Sigma}_1$)

Δ	Parametric			Non-parametric				
	Global	Hotelling	Roast	P score	GSEA	N-Statistic	KS_mean	KS_var
0	0.047	0.046	0.050	0.054	0.049	0.055	0.045	0.047
0.1	0.089	0.069	0.120	0.045	0.115	0.091	0.049	0.044
0.3	0.447	0.293	0.510	0.080	0.516	0.436	0.111	0.062
0.5	0.827	0.580	0.869	0.280	0.872	0.825	0.256	0.122
0.7	0.952	0.757	0.978	0.700	0.975	0.954	0.514	0.187
0.9	0.994	0.837	0.996	0.952	0.995	0.994	0.731	0.251

Table S13: Proportion of rejection for each of the GSAs under the following distributions

Control group	Case group
MVT($\underset{\sim}{\mathbf{0}}_{30\times1}, \mathbf{\Sigma}_2$)	0.5MVT($\underset{\sim}{\mathbf{0}}_{30\times1}, \mathbf{\Sigma}_2$) + 0.5MVT($\underset{\sim}{\mathbf{\Delta}}_{30\times1}, \mathbf{\Sigma}_2$)

Δ	Parametric			Non-parametric				
	Global	Hotelling	Roast	P score	GSEA	N-Statistic	KS_mean	KS_var
0	0.057	0.048	0.052	0.055	0.060	0.054	0.050	0.035
0.1	0.070	0.057	0.069	0.044	0.076	0.075	0.063	0.040
0.3	0.194	0.112	0.193	0.083	0.196	0.282	0.219	0.071
0.5	0.458	0.248	0.455	0.240	0.469	0.633	0.518	0.104
0.7	0.676	0.386	0.672	0.549	0.679	0.836	0.736	0.164
0.9	0.853	0.563	0.851	0.789	0.864	0.957	0.888	0.213

Table S14: Proportion of rejection for each of the GSAs under the following distributions

Control group	Case group
MVN($\underset{\sim}{\mathbf{0}}_{30\times1}, \mathbf{\Sigma}_1$)	0.4MVN($\underset{\sim}{\mathbf{0}}_{30\times1}, \mathbf{\Sigma}_1$) + 0.3MVN($0.5\times\underset{\sim}{\mathbf{\Delta}}_{30\times1}, \mathbf{\Sigma}_1$) + 0.3MVN($\underset{\sim}{\mathbf{\Delta}}_{30\times1}, \mathbf{\Sigma}_1$)

Δ	Parametric				Non-parametric			
	Global	Hotelling	Roast	P score	GSEA	N-Statistic	KS_mean	KS_var
0	0.043	0.036	0.044	0.054	0.043	0.040	0.040	0.046
0.1	0.084	0.060	0.096	0.047	0.104	0.081	0.066	0.035
0.3	0.345	0.207	0.417	0.071	0.421	0.339	0.078	0.053
0.5	0.701	0.469	0.793	0.166	0.792	0.691	0.198	0.087
0.7	0.938	0.732	0.967	0.493	0.967	0.941	0.392	0.161
0.9	0.986	0.853	0.992	0.840	0.992	0.985	0.599	0.228

Table S15: Proportion of rejection for each of the GSAs under the following distributions

Control group	Case group
MVT($\underset{\sim}{\mathbf{0}}_{30\times1}, \mathbf{\Sigma}_2$)	0.4MVT($\underset{\sim}{\mathbf{0}}_{30\times1}, \mathbf{\Sigma}_2$) + 0.3 MVT($0.5\times\underset{\sim}{\mathbf{\Delta}}_{30\times1}, \mathbf{\Sigma}_2$) + 0.3 MVT($\underset{\sim}{\mathbf{\Delta}}_{30\times1}, \mathbf{\Sigma}_2$)

Δ	Parametric				Non-parametric			
	Global	Hotelling	Roast	P score	GSEA	N-Statistic	KS_mean	KS_var
0	0.048	0.042	0.047	0.054	0.041	0.043	0.037	0.053
0.1	0.076	0.053	0.074	0.047	0.079	0.074	0.070	0.047
0.3	0.185	0.109	0.191	0.070	0.196	0.248	0.199	0.068
0.5	0.388	0.190	0.377	0.150	0.390	0.505	0.405	0.070
0.7	0.602	0.339	0.610	0.400	0.622	0.781	0.676	0.144
0.9	0.777	0.445	0.774	0.648	0.789	0.923	0.825	0.151
