# Supplementary material for: The misuse of distributional assumptions in functional class scoring gene-set and pathway analysis
Source: G3 (Bethesda). 2021 Oct 25;12(1):jkab365. doi: 10.1093/g3journal/jkab365 (PMC8728032; doi:10.1093/g3journal/jkab365)
Supplement: jkab365_Supplementary_FileS1 [file jkab365_supplementary_files1.pdf]

Supplementary File S1

Background information of the six MVN tests

Among the six MVN tests considered in this research, the Mardia test examines the normality assumption based on skewness and kurtosis (Mardia 1970). It is usually referred to as the Mardia skewness test or Mardia kurtosis test, representing its natural extension of univariate skewness and kurtosis tests to the multivariate case. It is therefore not robust to other types of deviations from normality and its power is unstable against many alternatives (Thode 2002; Mecklin and Mundfrom 2005; Zhou and Shao 2014; Chen and Xia 2019). The Henze-Zirkler (HZ) test evaluates the weighted distance between the observed multi-dimensional data and the multivariate normal distribution (Henze and Zirkler 1990). The distance is usually defined as the squared difference between two corresponding Fourier transformations (characteristic functions) of the observed and expected observations, and then weighted by a kernel function.

The Royston test (Royston 1992) applies the Shapiro-Wilk (SW) statistic (Shapiro and Wilk 1965), a univariate normality test utilizing variance ratios of the order statistics of observed data, to each coordinate of random vectors, and then combines the marginal statistics. In contrast to this, alternative methods focus on transforming the multivariate data into a univariate value via projection, since it is well known that a *p*-variate random vector $\mathbf{X}$ is normal if and only if $\theta\mathbf{X}$ is univariate normal for all $\theta \in \left\{\theta \in R^p : \|\theta\| = 1\right\}$. Following this rationale, the Fattorini (FA) test (Fattorini 1986; Lee *et al*. 2014) and TN test (Zhou and Shao 2014) were proposed based on the projection of $\mathbf{X}$ and SW statistics, respectively. Another test not in the previous categories is a nonparametric test, the Energy test (Székely and Rizzo 2005). It

defines the “energy” as a function of the distance between the data and the null multivariate normal distribution, and then examines if this energy statistic achieves statistical significance.

Based on simulation studies, Mecklin and Mundfrom (2005) recommend the HZ and Royston tests based on their stable type I and II error rates. Korkmaz, Goksuluk, and Zararsiz (2014) suggest the HZ test if the sample size is larger than 100, and the Royston test if smaller than 50. The simulation studies in Székely and Rizzo (2005) compared the EN test with the HZ and Mardia tests and concluded that the Energy and HZ tests have comparable performance. The TN test performs better than FA and HZ when the alternative hypothesis is a mixture of normal, chi-square or gamma distributions (Zhou and Shao 2014). Note that these results and conclusions are based on simulated observations from lower dimensional distributions and a large ratio of sample size to dimension (Mecklin and Mundfrom 2005; Székely and Rizzo 2005; Korkmaz *et al*. 2014), which may not reflect the case of a gene set containing a large number of genes.

References

Chen, H., and Y. Xia, 2019 A nonparametric normality test for high-dimensional data. arXiv: 1904.05289 [stat.ME].

Fattorini, L., 1986 Remarks on the use of Shapiro-Wilk statistic for testing multivariate normality. Statistica 46:209–217.

Henze, N., and B. Zirkler, 1990 A class of invariant consistent tests for multivariate normality. Commun Stat-A Theor. 19: 3595–3617.

Korkmaz, S., D. Goksuluk, and G. Zararsiz, 2014 MVN: An R package for assessing multivariate normality. R J. 6: 151–162.

Lee, R., M. Qian, and Y. Shao, 2014 On rotational robustness of Shapiro-Wilk type

tests for multivariate normality. Open J Stat. 2: 964–969.

Mardia, K.V., 1970 Measures of multivariate skewness and kurtosis with applications. Biometrika 57: 519–530.

Mecklin, C. J., and D. J. Mundfrom, 2004 An appraisal and bibliography of tests for multivariate normality. Int Stat Rev. 72: 123–138.

Mecklin, C. J., and D. J. Mundfrom, 2005 A Monte Carlo comparison of the type I and type II error rates of tests of multivariate normality. J Stat Comput Sim. 75: 93–107.

Royston P. 1992 Approximating the Shapiro-Wilk W test for non-normality. Stat Comput. 2:117–119.

Székely, G. J., and M. L. Rizzo, 2005 A new test for multivariate normality. J Multivar Anal. 93: 58–80.

Thode, H. C. Jr., 2002 Testing for normality. New York: Marcel Decker, Inc.

Zhou, M., and Y. Shao, 2014 A powerful test for multivariate normality. J Appl Stat. 41: 351–363.
